# Comparison of Extraction Methods for the Detection of Avian Influenza Virus RNA in Cattle Milk

**DOI:** 10.3390/v16091442

**Published:** 2024-09-10

**Authors:** Chantal J. Snoeck, Aurélie Sausy, Manon Bourg, Judith M. Hübschen

**Affiliations:** 1 Clinical and Applied Virology Group, Department of Infection and Immunity, Luxembourg Institute of Health, L-4354 Esch-sur-Alzette, Luxembourg; 2 Laboratoire de Médecine Vétérinaire de l’Etat, Luxembourg Veterinary and Food Administration (ALVA), L-3555 Dudelange, Luxembourg

**Keywords:** dairy cattle, milk, highly pathogenic avian influenza, H5N1, RNA extraction, PCR

## Abstract

Since early 2024, a multistate outbreak of highly pathogenic avian influenza H5N1 has been affecting dairy cattle in the USA. The influenza viral RNA concentrations in milk make it an ideal matrix for surveillance purposes. However, viral RNA detection in multi-component fluids such as milk can be complex, and optimization of influenza detection methods is thus required. Raw bulk tank milk and mastitis milk samples were artificially contaminated with an avian influenza strain and subjected to five extraction methods. HCoV-229E and synthetic RNA were included as exogenous internal process controls. Given the high viral load usually observed in individual raw milk samples, four out of five tested methods would enable influenza detection in milk with normal texture, over a time window of at least 2 weeks post-onset of clinical signs. Nevertheless, sample dilution 1:3 in molecular transport medium prior to RNA extraction provided the best results for dilution of inhibitory substances and a good recovery rate of influenza RNA, that reached 12.5 ± 1.2% and 10.4 ± 3.8% in two independent experiments in bulk milk and 11.2 ± 3.6% and 10.0 ± 2.9% on two cohorts of mastitis milk samples. We have also shown compatibility of an influenza RT-qPCR system with synthetic RNA detection for simultaneous validation of the RNA extraction and RT-qPCR processes.

## 1. Introduction

Starting in late January 2024, unexplained decreases in milk production and production of abnormal milk, associated with decreased feed intake, were observed in dairy cattle in Texas [1], as well as Kansas and New Mexico [2]. The etiological agent had been officially identified as highly pathogenic avian influenza (HPAI) H5N1 by the United States Department of Agriculture by the end of March 2024 [3], constituting the first documented cases of HPAI in cattle (reviewed in [4]). By August 13th, H5N1 had been identified in 190 dairy farms in 13 States in the United States of America (USA) [5]. The outbreaks are caused by genotype B3.13, originating from several successive reassortments between clade 2.3.4.4b H5N1 strains introduced from Europe to North America in 2021 [6] and low pathogenic strains circulating in wild birds in North America [7]. Genotype B3.13 has been detected in wild birds in 2023 in the USA and phylogenetic analyses suggested that HPAI H5N1 circulation in cattle resulted from a single bird-to-cattle spillover event estimated to have happened between November 2023 and January 2024 [1,7,8]. Following the species jump, movements of infected animals contributed to genotype B3.13’s spread across states [1,9,10]. Other factors such as sharing equipment, personnel, milk tankers and deadstock removal haulers may have further contributed to spread between premises [10,11]. H5N1 was also transmitted from cattle to cats, peri-domestic mammals and birds, poultry [1,2,11,12] as well as humans with occupational exposure to cattle [13,14]. These events highlight the need for integrated approaches to limit the spread to new areas and multiple species.

In cattle, the main reported signs of H5N1 infections were decreased and abnormal milk production (colostrum-like appearance, thickness, curdling), decreased feed consumption, dry feces or diarrhea, lethargy, dehydration, fever and mild respiratory signs [2,15,16]. Most animals recover within 2 to 3 weeks of clinical onset and the overall low morbidity and mortality in dairy cattle [2,12] contrasts with the situation in highly susceptible poultry. This, coupled with the high economic value of the animals, requires containment measures alternative to depopulation, such as restricted movement and enhanced testing [17,18]. Adaptation of surveillance schemes and detection methods are therefore needed for the surveillance of IAV in cattle.

H5N1 viral ribonucleic acid (RNA) has been detected in various specimens, including nasal swabs, blood or serum, but the highest viral loads were observed in milk samples [2], likely contributing to lateral spread through the contamination of milking material on which influenza A virus (IAV) can remain infectious for at least a few hours [19]. As measured by reverse transcription quantitative real-time PCR (RT-qPCR), quantification cycles (Cq) as low as 8–10 have been detected in milk of cattle with clinical signs [2] and remained detectable for at least 2–3 weeks post-disease onset [2,20]. Milk thus appears to be a suitable matrix for IAV detection upon disease suspicion. It has also been used as matrix for pre-movement testing [17], and retail dairy products have been screened to gain an overview of virus dissemination. IAV RNA has been detected in 10–23% of retail milk with various level of fat content in the USA, as well as in other dairy products such as butter, ice cream or cheese [21,22], but not in Canada [23,24,25]. Since no infectious virus was retrieved from retail dairy samples which tested positive [21,22], viral RNA detection by RT-qPCR may be used as a fast and sensitive method for testing individual milk samples as well as bulk retail items. Traditionally, virus detection in complex multi-component fluids such as milk is more difficult, compared to samples such as swab supernatant, and needs to be optimized. In particular, various degrees of milk dilution prior to RNA extraction to decrease the effect of inhibitory substances have been applied in some studies [23] or recommended by reference organizations [26,27], while others apply standard protocols without any pre-processing [2,21,25], but no formal comparison of such methods have been provided so far. In anticipation of a potential international spread of genotype B3.13 or local clade 2.3.4.4b spillover to cattle in Europe [28], we evaluated different extraction methods for the detection of IAV RNA in raw milk. Since viral RNA detection in the milk of both asymptomatic and symptomatic animals has been described, we assessed the methods on both raw bulk tank milk and raw milk from animals with mastitis, which was artificially contaminated with an IAV strain.

## 2. Materials and Methods

### 2.1. Milk Samples

Fresh raw bulk tank milk was purchased from a local farm and kept refrigerated until use. Leftovers of individual raw milk samples from animals with mastitis submitted for microbiology analyses were kindly provided by the Laboratoire de Médecine vétérinaire de l’Etat. They were also kept refrigerated until use.

### 2.2. Amplification of Virus Stocks

A low pathogenic H6N2 influenza A strain was used as a surrogate to HPAI H5N1 for all experiments. The strain A/mallard/Germany/R1501/08, kindly provided by the Friedrich-Loeffler-Institut in Germany, was propagated on Madin-Darby canine kidney (MDCK) cells (ATCC CCL-34, LGC Standards, Molsheim, France). Briefly, MDCK cells were maintained in DMEM (Gibco, Merelbeke, Belgium), supplemented with 10% fetal bovine serum (Gibco), 1% penicillin-streptomycin (Capricorn, Leusden, The Netherlands), 0.2% bovine serum albumin (Gibco) and HEPES buffer (Gibco). Cell monolayers confluent at 80% were rinsed with phosphate buffer saline before inoculation of the influenza A strain at a 1:2 dilution (500 µL of virus + 1000 µL of virus growth medium). The 175 cm^2^ flask was incubated for 30 min at 37 °C and with 5% CO_2_ to allow binding of viral particles to cellular receptors prior to adding virus growth medium composed of DMEM supplemented with 6-(1-tosylamido-2-phenyl) ethyl chloromethyl ketone (TPCK)-treated trypsin (Merck, Overijse, Belgium), 1% penicillin-streptomycin, 0.2% bovine serum albumin and HEPES. Cells were incubated at 37 °C with 5% CO_2_ and monitored daily for the presence of cytopathogenic effect. When cytopathogenic effect reached 60% to 70%, flask content was aspirated, and cellular debris were removed by centrifugation. Virus stocks were supplemented with bovine serum albumin to reach a final concentration of 0.5%, aliquoted and stored at −80 °C. Quantification of the virus stock, in genome copies (gc)/mL, was performed by IAV-1 reverse transcription quantitative real-time PCR (RT-qPCR) by including a tenfold dilution series of quantitative genomic RNA (VR-1894DQ, LGC Standards).

Human coronavirus 229E (HCoV-229E), acquired from Culture Collections, UK Health Security Agency (Salisbury, UK; Cat. No. 2008101v), was used as an exogenous internal process control (IPC) after propagation on MRC-5 cells (European Collection of Authenticated Cell Cultures, Salisbury, UK; Cat. No. 05072101). Briefly, MRC-5 cells were maintained in DMEM low glucose supplemented with L-glutamine and sodium pyruvate (Gibco), 1% non-essential amino acids (Life Technologies, Merelbeke, Belgium), 10% fetal bovine serum and 1% penicillin-streptomycin. When 80% confluence was reached, cell monolayers were rinsed with phosphate buffer saline before inoculation with HCoV-229E. Cells were incubated for 60 min at 33 °C with 5% CO_2_ before adding virus growth medium (same composition as cell maintenance medium, with fetal bovine serum concentration reduced to 2%). Daily monitoring and harvesting was similar to the procedure described for the IAV strain. Quantification of the viral stock was performed by RT-qPCR, by including a tenfold dilution series of quantitative genomic RNA (VR-740DQ, LGC Standards) in triplicates.

### 2.3. Artificial Sample Contamination

First, 300 µL of a tenfold serial dilution of H6N2 cell culture supernatant was added to 2.7 mL bulk milk aliquots or virus transport medium (VTM) as a reference. Final IAV concentrations were selected to cover the lower range of IAV RNA concentrations detected in infected cattle [2,20] or in milk products [21] and ranged from 8.2 × 10^3^ to 8.2 × 10^6^ gc/mL. All contaminated samples were generated in triplicate and extracted separately (technical triplicates) according to different methods (see below).

Secondly, the different methods were tested on a first panel of six individual milk samples from cows presenting signs of mastitis (thereafter referred to as “mastitis milk samples”) and compared to virus transport medium (in triplicates) and bulk milk (in triplicates). All samples (3 mL) were seeded with 30 µL of H6N2 cell culture supernatant, corresponding to a final concentration of 8.2 × 10^5^ gc/mL. The most promising method was selected and evaluated on a second panel of 12 individual mastitis milk samples, in comparison with virus transport medium (in triplicates) and bulk milk (in triplicates). This last sample panel was also used to evaluate an additional RNA extraction method. Each panel of mastitis milk contained samples with visible curdling and discoloration.

### 2.4. Viral RNA Extraction

**Figure 1:**
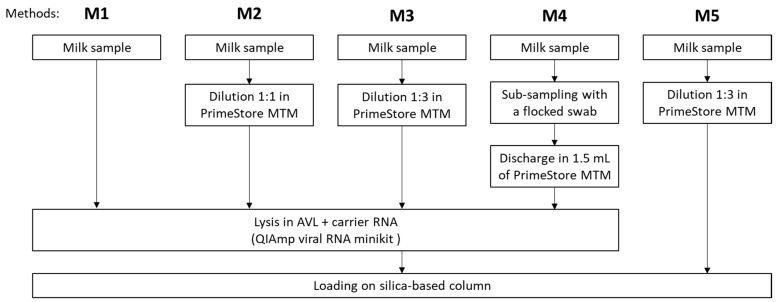
**Schematic illustration of the five methods compared for viral RNA extraction of IAV in cattle milk.** Method M1 corresponds to the manufacturer’s instructions with RNA extraction directly from 140 µL of medium or milk. For methods M2 or M3, milk samples were diluted 1:1 (method M2) or 1:3 (method M3) in molecular transport medium prior to RNA extraction from the diluted sample. For method M4, a swab was dipped into undiluted milk prior to being discharged in molecular transport medium. RNA was extracted from the molecular transport medium. Finally, for method M5, milk was diluted 1:3 in molecular transport medium prior to RNA extraction omitting adding lysis into AVL and directly loading on the silica-based membrane spin column.

Viral RNA from all samples was extracted with the QIAamp Viral RNA minikit (Qiagen, Venlo, The Netherlands) according to different pre-processing steps. Method 1 (M1) corresponded to the manufacturer’s instructions; i.e., viral RNA was directly extracted from 140 µL of medium or raw milk (Figure 1). Molecular transport medium (PrimeStore MTM, Longhorn, Bethesda, MD, USA), containing guanidine thiocyanate, ethanol, and N-Lauroylsarcosine sodium, has been shown to inactivate viruses, including IAV, while stabilizing nucleic acids for subsequent molecular detection [29]. PrimeStore MTM has also been recommended for milk dilution prior to RNA extraction [27]. Therefore, milk samples were diluted 1:1 (method M2) or 1:3 (method M3) in molecular transport medium. After homogenization, 140 µL of diluted milk samples were used for RNA extraction. To ease sample dispatch, distribution of PrimeStore MTM collection tubes directly to the farms to allow on-site milk subsampling with a swab was proposed [27]. For method 4 (M4), a flocked nylon-fiber swab (Copan, Brescia, Italy) was dipped into undiluted contaminated milk samples and swirled for 30 s. Swabs were then transferred into a tube containing 1.5 mL of molecular transport medium, swirled, pressed against the tube wall and discarded. The volume of milk absorbed on a swab was estimated by measuring the mass lost after swirling a swab in 1 mL of bulk milk (*n* = 10) with a measured density of 1.028 g/mL. The volume absorbed corresponded to 133 ± 5 µL, leading to a 1:11 dilution factor. After homogenization, 140 µL of the molecular transport medium/milk mixture was used for RNA extraction. Two types of exogenous IPCs were added to monitor the effect of pre-processing steps on inhibitors. HCoV-229E shares general characteristics with IAV, since both are enveloped viruses with single stranded RNA genomes. The use of a commercial IPC consisting of synthetic exogenous RNA was also assessed as a surrogate to HCoV-229E and for possible co-detection with IAV in duplex format. IPCs, i.e., 5 µL of HCoV-229E virus stock (corresponding to 6.8 × 10^3^ gc) and 2 µL (2 × 10^4^ copies) of commercial IPC (VetMAX Xeno Internal Positive Control RNA, Applied Biosystems, Merelbeke, Belgium), were added into the lysis buffer AVL for all samples except negative controls. As PrimeStore MTM is already an inactivating agent, method 5 (M5), which skips the lysis step from the extraction kit, was assessed on the second panel of mastitis milk samples. For this method, a final volume of 1260 µL of milk diluted 1:3 in molecular transport medium was spiked with 5 µL of HCoV-229E virus stock and 2 µL of commercial IPC, and directly loaded on the spin columns, omitting the lysis step from the kit. All samples were eluted in 60 µL of elution buffer.

### 2.5. Reverse Transcription Quantitative Real-Time PCR (RT-qPCR)

HCoV-229E detection made use of the forward 5′-CAGTCAAATGGGCTGATGCA-3′ and reverse 5′-AAAGGGCTATAAAGAGAATAAGGTATTCT-3′ primers and 5′-[FAM]-CCCTGACGACCACGTTGTGGTTCA-[BHQ-1]-3′ probe [30]. The reactions were carried out with a QuantiTect Probe RT-PCR Kit (Qiagen) in a 25 µL final volume containing 500 nM of each HCoV-229E primer, 100 nM of HCoV-229E probe (Kaneka Eurogentec, Liège, Belgium) and 1 µL of VetMAX Xeno Internal Positive Control—VIC Assay (Applied Biosystems) primer mix. Cycling conditions were as follows: reverse transcription at 50 °C for 30 min and 95 °C for 15 min, followed by 45 cycles at 95 °C for 15 s and 60 °C for 1 min.

Influenza A viral RNA was detected using an RT-qPCR targeting the matrix gene, shown to broadly detect IAV strains from avian, swine and human origin ([31], assay thereafter referred to as “IAV-1”) and approved as a diagnostic method for IAV in the European Union [32]. The reaction was performed in a 25 µL volume using QuantiTect Probe RT-PCR Kit (Qiagen), 600 nM of forward 5′-GGCCCCCTCAAAGCCGA-3′ and reverse 5′-CGTCTACGYTGCAGTCC-3′ primers (Kaneka Eurogentec) and 212 nM of 5′-[FAM]-TCACTKGGCACGGTGAGCGT-[MGB]-3′probe (Life Technologies). Cycling conditions were as follows: reverse transcription at 50 °C for 30 min and 95 °C for 15 min, followed by 45 cycles at 95 °C for 10 s, 64 °C for 30 s and 72 °C for 10 s.

Since IAV-1 is not compatible with duplex detection of the commercial IPC due to the high annealing temperature, we investigated the use of a second universal IAV detection RT-qPCR also targeting the matrix gene [33] (hereafter referred to as “IAV-2”), also approved as diagnostic method in the European Union [32]. The assay includes one forward primer 5′-AGATGAGYCTTCTAACCGAGGTCG-3′, four reverse primers 5′-TGCAAA[A/T/C/G]ACATCYTCAAGTCTCTG-3′, with each individual primer having one of the four bases in position 7, and the probe 5′-[FAM]-TGCAAAAACATCYTCAAGTCTCTG-[TAMRA]-3′. For amplification, the AgPath-ID One-Step RT-PCR chemistry (Applied Biosystems) with 900 nM of forward primer, 225 nM of each of the four reverse primers, and 250 nM of the probe (Kaneka Eurogentec) with (IAV-2 duplex) or without (IAV-2 singleplex) 1 µL of VetMAX Xeno Internal Positive Control—VIC Assay primer mix was used. Cycling conditions were as follows: reverse transcription at 45 °C for 10 min and 95 °C for 10 min, followed by 45 cycles at 95 °C for 15 s and 60 °C for 45 s. Compatibility of dual detection was checked by amplifying a tenfold dilution of A/Cambodia/E0826360/2020 (H3N2; virus isolate Cat. No. 20/310, procured from the National Institute for Biological Standards and Control, South Mimms, UK) RNA extracted with method M1 with IAV-2 singleplex or duplex RT-qPCR.

All reactions were performed in 25 µL volumes with 5 µL of RNA on a CFX96 Touch (IAV-1) or CFX Opus 96 (HCoV-229E duplex, IAV-2 in singleplex or duplex; BioRad, Temse, Belgium) real-time instruments. Fluorescence was measured in the FAM (singleplex) or FAM and VIC (duplex) channels. Data analysis was performed with CFX Maestro v1.1 Software (BioRad). IAV RNA quantification was performed by including a tenfold dilution series of quantitative genomic RNA (VR-1894DQ, LGC Standards) in triplicate in each assay.

### 2.6. Data Analysis

Samples with no fluorescence signal were attributed a Cq value of 45 and a viral load of 1 gc/µL for graphical display. Viral load in contaminated samples, expressed in gc/µL of RNA extraction eluate, was determined based on values extrapolated from standard curve data. Standard curve data respected qPCR requirements; i.e., they had efficiency between 90 and 105% and R^2^ > 0.98. Recovery rates (in %) were calculated as the viral load in a sample divided by the average viral load obtained with virus transport medium with method M1, multiplied by 100. Samples with no fluorescence signal for HCoV-229E or commercial IPC were attributed a Cq of 45 for calculation of mean Cq. Comparison of inhibitor levels according to RNA extraction methods were performed by calculating the difference between the average Cq in samples extracted with each method and the average Cq observed in VTM with method M1 (referred to as ∆Cq). Graphic representation was performed in GraphPad Prism v10.1.2 (Dotmatics, Boston, MA, USA).

## 3. Results

### 3.1. Comparison of Extraction Methods Using Bulk Tank Milk

For the initial method comparison, bulk tank milk samples were artificially contaminated with tenfold dilutions of IAV. Two types of internal process controls—HCoV-229E sharing characteristics with IAV and a commercial control for potential duplex detection—were added to monitor the effect of pre-processing steps on inhibitors.

**Figure 2:**
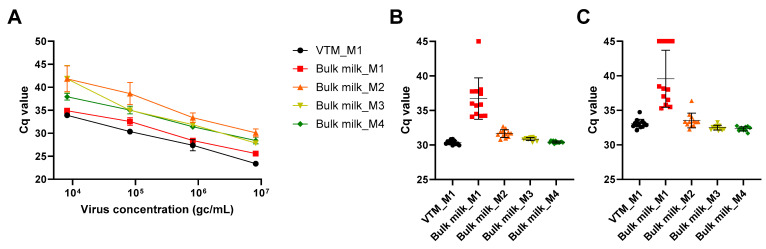
**IAV and IPC detection in virus transport medium (VTM) and raw bulk tank milk.** Triplicate VTM and bulk milk samples were artificially contaminated with four ten-fold dilutions of IAV and extracted with methods M1 to M4. Constant quantities of HCoV-229E and commercial IPC were added at the lysis step. (**A**) Cq values obtained with IAV-1 RT-qPCR (y-axis) corresponding to final IAV concentrations ranging from 8.2 × 10^3^ to 8.2 × 10^6^ gc/mL of milk (x-axis). Mean values and standard deviations from three technical replicates are shown. (**B**,**C**) Cq values from RT-qPCR detecting both hCoV-229E (**B**) and commercial IPC (**C**) in a duplex format. The results of samples contaminated with four IAV dilutions in three technical replicates each are shown. In the absence of inhibition, Cq values for HCoV-229E or commercial IPC are expected to be similar across RNA extraction methods.

**Figure 3:**
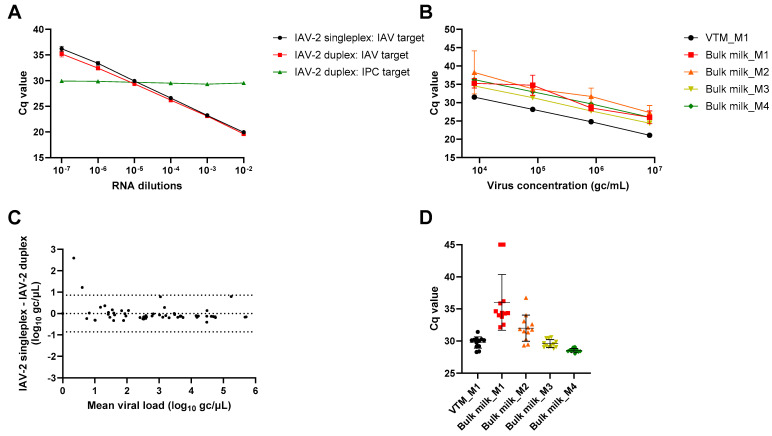
**Assessment of compatibility of IAV and commercial IPC detection in IAV-2 duplex RT-qPCR system.** (**A**) Tenfold dilutions of A/Cambodia/E0826360/2020 (H3N2) viral RNA were tested in technical triplicates in mastermix containing no IPC and no IPC detection primers and probe (IAV target, black symbols), and mastermix containing both a constant quantity of IPC RNA (1 × 10^3^ copies) and IPC detection primers and probe (IAV target, red symbols). Detection of IPC in the VIC channel in duplex mastermix is shown with the green symbols (IPC target). Mean Cq values and standard deviations from three technical replicates are shown. (**B**) Cq values obtained with IAV-2 singleplex RT-qPCR for samples artificially contaminated with four ten-fold dilutions of IAV and extracted with methods M1 to M4 (same as for Figure 2A). Mean Cq values and standard deviations from three technical replicates are shown. (**C**) Bland–Altman plot of viral load (in log_10_ gc/µL) measured with IAV-2 RT-qPCR in singleplex or duplex. Average mean of all differences and 95% limits of agreement are shown with dotted lines. (**D**) Cq values of detection of commercial IPC with IAV-2 duplex RT-qPCR. The results of three technical replicates of samples contaminated with four IAV dilutions are shown. Lower Cq values are systematically obtained for commercial IPC with IAV-2 duplex compared to duplex detection with HCoV-229E (Figure 2C), due to higher sensitivity of IAV-2 RT-qPCR chemistry for amplification of the commercial IPC.

With method M1 used as a reference, IAV detection in bulk milk showed higher Cq values compared to virus transport medium regardless of the viral concentration artificially seeded in the samples (Figure 2A). Higher Cq values were also obtained with pre-processing methods M2–M4 compared to method M1 in bulk milk. Interestingly however, higher degrees of bulk milk dilution in molecular transport medium (methods M3 and M4) had lower Cq values compared to the 1:1 dilution (method M2). In parallel, variable levels of inhibition were detected in bulk milk compared to virus transport medium, with both HCoV-229E and commercial IPC as controls (Figure 2B,C). Indeed, ∆Cq were 6.35, 1.29, 0.50 and 0.06 for HCoV-229E in milk samples extracted with methods M1, M2, M3 and M4 respectively. For the commercial IPC, ∆Cq were 6.50, 0.49, −0.58 and −0.75 in milk samples extracted with methods M1, M2, M3 and M4 respectively. Thus, bulk milk dilution with methods M2–M4 seemed to gradually decrease inhibitor levels, helping to recover IAV signal despite higher sample dilutions in methods M3–M4 compared to method M2. Similar results were observed when IAV detection was carried out using IAV-2 RT-qPCR (Figure 3). The main difference resided in the overall lower Cq values obtained with IAV-2 RT-qPCR (Figure 3B). Higher Cq values in bulk milk extracted with method M1 compared to method M3 were also obtained, which may indicate slightly higher sensitivity of the IAV-2 RT-qPCR chemistry towards inhibitors present in the milk matrix. Comparison of IAV RNA detection in IAV-2 singleplex and duplex formats showed similar results (Figure 3C), while the IAV-2 duplex assay also allowed the concomitant assessment of the presence of inhibitory substances (Figure 3D). Indeed, ∆Cq were 6.25, 2.23, −0.17 and −1.22 in milk samples extracted with methods M1, M2, M3 and M4 respectively. IAV RNA detection was thus performed with IAV-2 duplex RT-qPCR for the subsequent experiments.

### 3.2. Comparison of Extraction Methods Using Individual Milk Samples

Since abnormal milk production is commonly reported in HPAI H5N1 affected cattle [2,11], we compared the different extraction methods using individual milk samples originating from cows with mastitis, including some samples with visible discoloration and curdling.

**Figure 4:**
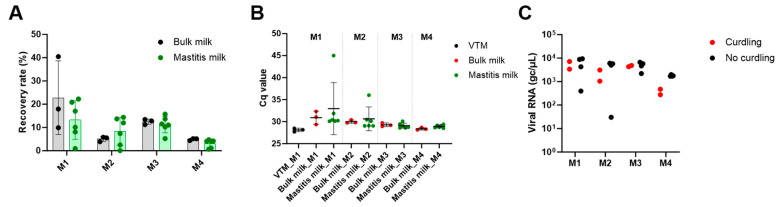
**IAV and IPC detection in bulk milk and individual milk samples from cows with mastitis.** A single IAV concentration was seeded into VTM (technical replicates, *n* = 3), bulk milk (technical replicates, *n* = 3) and mastitis milk samples (biological replicates, *n* = 6), while a constant concentration of commercial IPC was added at the lysis step. IAV detection was performed with IAV-2 duplex RT-qPCR. (**A**) Recovery rates, calculated as the ratio of viral loads in a sample extracted with methods M1–M4 to the average viral load in VTM with method M1. Mean values and standard deviations of the replicates are shown. (**B**) Cq values (and mean and standard deviations) for commercial IPC detection with IAV-2 duplex RT-qPCR are shown. (**C**) Viral loads measured in individual mastitis samples extracted with methods M1–M4 are displayed, with samples with curdling highlighted in red.

Mean recovery rates in bulk milk ranged from 22.7 ± 15.8% with method M1, 5.1 ± 1.0% with method M2, 12.5 ± 1.2% with method M3 and 4.9 ± 0.4 with method M4 (Figure 4A). Mean recovery rates in mastitis milk samples ranged from 13.2 ± 8.3% with method M1, 8.3 ± 6.0% with method M2, 11.2 ± 3.6% with method M3 and 3.1 ± 1.8% with method M4, with high variability between samples. Lower variability was observed with methods M3 and M4, likely owing to the reduced level of inhibitors (Figure 4B). Indeed, ∆Cq compared to VTM were 2.44, 2.52, 0.98 and 0.81 in mastitis milk samples extracted with methods M1, M2, M3 and M4 respectively. Despite low levels of inhibitors released in the medium with method M4, in practice we observed that swabs swirled in samples with visible curdling were coated with fat, and swab homogenization in molecular transport medium was suboptimal. This was also reflected in the lower IAV viral load detected for such samples (Figure 4C). This suggested that subsampling of milk with abnormal consistency with a swab may have a reduced efficiency, and this option was thus no longer considered. Similarly, method M2 was no longer considered, owing to the lower recovery rates and higher degree of variability compared to methods M1 and M3 (Figure 4).

**Figure 5:**
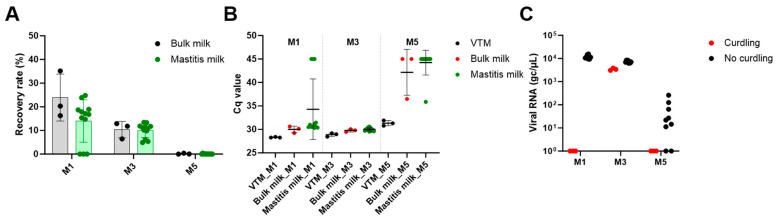
**IAV and IPC detection in a second set of individual milk samples from cows with mastitis.** A single IAV concentration was seeded into VTM, bulk milk and individual mastitis milk samples (*n* = 12) while constant concentration of commercial IPC was added at the lysis step. IAV detection was performed with IAV-2 duplex RT-qPCR. (**A**) Recovery rates in bulk milk (three technical replicates) and mastitis milk samples (12 biological replicates) obtained with methods M1, M3 and M5 are shown. Mean values and standard deviations of the replicates are shown. (**B**) Cq values (and mean and standard deviations) for commercial IPC detection with IAV-2 duplex RT-qPCR are shown (**C**). Viral loads measured in individual mastitis samples extracted with methods M1, M3 and M5 are displayed, with samples with curdling highlighted in red.

The experiment was repeated independently with a second set of individual mastitis milk samples. Mean recovery rates were 23.9 ± 9.9% and 14.0 ± 8.9% in bulk milk and mastitis milk samples, respectively, with method M1, and 10.4 ± 3.8% and 10.0 ± 2.9% with M3 (Figure 5A). The beneficial effect of dilution (method M3) for milk samples with visible curdling was again shown. Indeed, neither influenza RNA nor the process control were detected for the 3 out of 12 samples with abnormal consistency using method M1 (Figure 5B,C), while the signals were recovered upon dilution (M3). As PrimeStore MTM is already an inactivating agent, we further tested the possibility of omitting additional lysis with the lysis buffer provided in the kit (method M5) to decrease turn-around times. This method, however, did not show satisfactory results. Already, when applied to artificially contaminated VTM, a mean 8-fold reduction of IAV viral load and IPC recovery compared to method M1 was observed. Recovery rates (compared to M1) were 0.15 ± 0.2% in bulk milk and 0.07 ± 0.1% in mastitis milk samples (Figure 5).

## 4. Discussion

Evidence of IAV in cattle was already available as early as the 1950s (reviewed by [4]), and most studies described seroconversion of cattle to human H1N1 or H3N2 strains [34,35,36,37]. Nevertheless, no long-term sustained cattle-to-cattle transmission of IAV strains has been documented, and IAV detection in milk is unprecedented, despite previous evidence of IAV seroconversion associated with a drop in milk yield [34,35,36]. Infection of the mammary gland currently seems key for sustaining viral replication and transmission in cattle. Indeed, experimental intranasal infection of dairy calves with HPAI H5N1 only led to transient infection, with no to little clinical signs [20,38] and limited evidence of transmission to contact calves [38]. Experimental infection with a genotype B3.13 strain through the teats of lactating cows, however, reproduced the disease course described in the field with high viral shedding in milk [20].

Prior to the current events, H5N1 goose/Guangdong lineage strains had been circulating for over two decades without adapting to cattle. It is possible that the particular genetic composition of genotype B3.13 and/or specific events leading to exposure of cattle mammary glands to HPAI H5N1 led to the current outbreak in the USA. International trade of animals or animal products may result in genotype B3.13’s introduction to cattle herds elsewhere. Canada is the main importer of live cattle from the USA, and restrictions, pre-movement testing and active surveillance are already ongoing [23,24,25]. Alternatively, HPAI H5N1 clade 2.3.4.4b strains have been circulating in wild birds almost worldwide since the 2022–2023 season and other clade 2.3.4.4b strains circulating in Europe interestingly seem to be able to replicate in cow udders too [28]. Bird-to-cattle transmission may not be an isolated event in the future, and the European Food Safety Authority (EFSA) recently recommended considering IAV in the differential diagnosis of unresolved disease manifestation in cattle, including severe drops in milk production and abnormal milk, as well as to expand testing to ruminants when HPAI H5N1 is circulating nearby [39].

Milk is the sample of choice for HPAI H5N1 diagnosis in lactating cattle, as other samples, such as nasal swabs, rectal swabs, feces, urine or blood samples were only occasionally positive in infected animals [2,20]. Documented viral concentrations in individual milk samples and udder of infected cattle were relatively high. Cq values in milk samples as low as 8–10 were reported, although variations between animals are noticeable and Cq up to 35 were also detected [2]. Milk samples in a farm in Ohio had a mean Cq of about 20 when collected 3 days post-clinical diagnosis and a mean Cq of about 31 after 16 days [2]. In experimental infection through the mammary gland, the peak of viral shedding was already reached 2 days post infection (dpi) with Cq of about 16–17. The Cq remained below 30 until 14 dpi and viral RNA remained detectable throughout the observation period (24 dpi). Viral load in milk was also correlated with the intensity of clinical signs, including rumen motility, milk production, milk discoloration and consistency [20]. Notification of clinical signs to the farm veterinarian is thus likely to occur early in the disease course, when viral shedding is at its peak, although movement of pre-clinical animals likely contributed to the geographic spread in the USA [1]. In this study, we showed IAV detection over a wide range of IAV concentrations seeded in bulk milk samples. Method M1 had the highest degree of variability. Similar observations were already made when this method was applied to raw goat milk artificially contaminated with a surrogate for tick-borne encephalitis virus [40], owing to the fact that various milk components interact differently with viral particles or viral RNA [41]. Nevertheless, extraction methods M1–M4 would in general enable the detection of IAV in milk with normal texture, over a time window of at least 2 weeks post-onset of clinical signs, given the range of observed Cq values in milk samples from infected cows. Bulk milk produced by many farms is usually pooled during milk processing in dairy plants, and milk from infected cattle may only represent a very low fraction of a single retail milk product. Nevertheless, Cq values ranging from 21 to 36 were obtained from retail fluid milk (0%, 1%, 2% fat and whole milk) products originating from several states in the USA [21]. Given the range of IAV concentrations included in our first tests (Figure 2A and Figure 3B), it is reasonable to assume that methods M1–M4 would also enable IAV RNA detection in such pooled milk products.

However, production of abnormal milk is one of the most frequently reported signs in infected cattle [2,11,20]. We thus aimed at verifying the compatibility of the methods in samples with unusual consistency. For this, two panels of samples originating from cows with mastitis were tested, including samples with obvious discoloration and curdling. Our results showed the presence of various levels of inhibitors in mastitis samples, resulting in more variable recovery rates between samples (Figure 4 and Figure 5) when no pre-processing steps are applied (method M1). This is particularly the case for milk samples in which fat clots likely decreased the efficiency of RNA extractions, possibly by obstructing the silica membrane. Similarly, fatty aggregates can stick to flocked swabs, preventing efficient release of the material in resuspension medium (method M4). Ultimately, method M3 showed the best results for dilution of inhibitory substances enabling more uniform recovery of IAV detection without decreasing the IAV signal, regardless of milk consistency. Method M3 also aligns with the manufacturer’s recommendation of sample dilution factor to ensure complete inactivation, although 1:1 ratios (method M2) were also shown to result in effective Eastern equine encephalitis virus/Sindbis chimera inactivation [29]. We have also shown compatibility of the IAV-2 RT-qPCR system with the detection of a commercial internal process control for simultaneous validation of the RNA extraction and RT-qPCR process, for efficient use of time and resources.

Although we investigated several methods of sample pre-processing, our study may have some limitations, in particular for not assessing additional intermediate dilution factors between 1:3 (method M3) and 1:11 (method M4). Sample dilution primarily aims to dilute inhibitory substances that alter RNA extraction and/or RT-qPCR efficiency. We showed that the 1:3 dilution (method M3) already allowed for the retrieval of Cq values of the internal process controls, similar to those obtained in virus transport medium both in bulk milk and in mastitis milk samples (Figure 2B,C, Figure 3D and Figure 4B), and comparable to the 1:11 dilution factor. Intermediate dilutions would thus reasonably provide a similar effect, while only diluting the IAV signal, since the retrieved viral load also decreased according to the dilution factor for bulk milk samples as well as the majority of mastitis samples with no evidence of curdling (Figure 4C and Figure 5C).

In conclusion, we investigated several extraction methods for the detection of IAV in milk, using a commercial kit associated with low levels of instrument needs/costs which can also be applied in settings with lower resources. Dilution in the inactivating agent would also allow safe and stable shipment at ambient temperature, further easing sample distribution to competent laboratories that may be located further away.

## Data Availability

The original contributions presented in the study are included in the article. Further inquiries can be directed to the corresponding author.
